# Feralgine™ a New Approach for Iron Deficiency Anemia in Celiac Patients

**DOI:** 10.3390/nu11040887

**Published:** 2019-04-20

**Authors:** Laura Giancotti, Valentina Talarico, Giuseppe Antonio Mazza, Santina Marrazzo, Pietro Gangemi, Roberto Miniero, Marco Bertini

**Affiliations:** 1 Department of Pediatrics, Pugliese-Ciaccio Hospital-Magna Graecia University, 88100 Catanzaro, Italy; lauragiancotti@virgilio.it (L.G.); talaricovalentina@gmail.com (V.T.); giuseppeantiom@gmail.com (G.A.M.); santina.marrazzo.ost@live.it (S.M.); roberto.miniero@unicz.it (R.M.); 2 Clinical Chemistry Laboratory, Pugliese-Ciaccio Hospital, 88100 Catanzaro, Italy; piero19532@virgilio.it; 3 R&D Department, Laboratori Baldacci SpA, 56124 Pisa, Italy

**Keywords:** oral iron absorption test, celiac disease, ferrous bisglycinate chelate alginate, iron deficiency anemia

## Abstract

Background: Celiac disease (CD) is an immunologically-mediated disorder characterized by duodenal mucosa villi atrophy. Iron absorption is usually reduced in celiac patients making every kind of oral iron treatment unhelpful because of malasorption. Feralgine™ is a new product that has been demonstrated to be more bioavailable. As such, the aim of our study was to evaluate the absorption of Feralgine™ in adult patients with CD. Methods: Twenty-six adults affected by Iron Deficiency Anemia (IDA), of which 14 were also affected by CD and 12 were not affected by CD, were enrolled. An oral iron absorption test (OIAT) was performed in each patient by administrating Feralgine™, and serum iron was evaluated at baseline (T0) and after 2 h (T1) from the oral iron ingestion. Results: The OIAT was well tolerated in all patients, and, surprisingly, an equivalent statistically significant improvement in serum iron occurred in the two groups of patients (IDA plus CD: T0 = 28.21 µg/dL vs. T1 = 94.14 µg/dL *p* = 0.004 and IDA without CD: T0 = 34.91 µg/dL vs. T1 = 118.83 µg/dL, *p* = 0.0003). Conclusions: These results demonstrated the high absorption of Feralgine™ in celiac patients, confirming our previous data obtained with Ferrous Bysglicinate in children with CD.

## 1. Introduction

Celiac disease (CD) is an immunologically-mediated enteropathy that develops in genetically susceptible individuals who, in response to unknown environmental factors, develop an immune response that is subsequently triggered by the ingestion of gluten [1]. It is characterized by small-bowel mucosal villous atrophy with crypt hyperplasia [2].

As iron absorption primarily occurs in the duodenum and the jejunum, and iron malabsorption is usually observed in CD. Iron deficiency anemia (IDA) is a frequent finding in patients with overt CD (10–20% of cases) [3]. A recent meta-analysis found that approximately 1 in 31 patients with IDA have histological evidence of CD. This high prevalence value justifies the practice of testing for CD the patients with IDA [4]. In many cases of IDA, a lack of response to therapy indicates an underlying celiac disease is present [5]. The treatment of IDA associated with CD is primarily a gluten-free-diet (GFD) and oral iron supplementation until the iron stores have been restored [6]. Recovery from anemia occurs between 6 and 12 months on GFD as a consequence of normalization of histological alterations of the intestinal mucosa; this process can take as long as two years for the iron stores to be replete [7]. The improvement in anemia could reflect the adherence to diet [8].

Ferrous sulfate remains the gold standard for oral iron supplementation in iron depleted patients, irrespective of the underlying disease [9,10]. The treatment is limited by the frequent gastrointestinal side effects, mainly due to the irritation and the chemical reactions with unabsorbed iron compounds [11]. A variety of other iron chemical forms are available; they are better tolerated but, unfortunately, less effective than ferrous sulfate. The poor tolerability and poor efficacy of oral iron preparations is of particular importance in patients with CD [12]. Ferrous bisglycinate chelate (FBC) is composed of an atom of ferrous iron that is chelated by two molecules of glycine through covalent and coordinate bonds [13]. It has been reported to be safe and effective in reversing IDA in adults, adolescents, and young children [14,15,16,17]. In order to improve the bioavailability, a new preparation of FBC named Feralgine^TM^ has been developed [18]. It is a patented co-processed one-to-one ratio compound between FBC and Sodium Alginate that uses spray-drying technologies, has been demonstrated to be a good oral treatment, is bioavailable, and has an optimal tolerability [18]. By applying spray-drying technologies to a solution of Ferrous Bisglycinate Chelate and Sodium Alginate, a new compound can be obtained in which Alginic Acid and Ferrous Bisglycinate Chelate are present in a one-to-one ratio and in which every little particle of the powder has the same morphology and quantity of the two different co-processed substances.

The oral iron absorption test (OIAT) is an easy to perform method that evaluates iron absorption and whether or not a patient with IDA will benefit from iron supplementation [19]. The test consists of measuring a plasma iron increase in the hours following a single dose of an oral iron preparation [20,21]. As such, we considered it interesting to use the OIAT to investigate the absorption of iron present in Feralgine^TM^ in adult patients with CD and IDA, as compared to non-celiac patients with IDA.

## 2. Patients and Methods

The study was an open prospective monocentric trial conducted at the Center for Diagnosis, Treatment and Follow-up of Celiac disease of the Magna Graecia University, Pugliese-Ciaccio Hospital, Catanzaro, Italy. A total of 26 consecutive adult patients with IDA were enrolled from January 2017 to October 2017. 14 (Group A) patients were newly diagnosed with CD (mean age: 32.28 years). 12 (Group B) patients were affected by IDA without CD or other gastrointestinal diseases (mean age: 33.58 years). The diagnosis of IDA was established according to the international accepted criteria considering serum iron, total iron-binding capacity, the tranferrin saturation index, serum ferritin, and red blood cell indices [9,22]. The diagnosis of CD was performed according to current guidelines for an adult subject: A positive response to circulating antibodies against tissue transglutaminase and with upper-gastrointestinal endoscopy and duodenal biopsies [1]. Any patient who had systemic chronic inflammation, renal insufficiency, active infections (an elevated value of C-reactive protein), or had been treated with chloramphenicol or other drugs that interfered with iron absorption was not taken into consideration.

The study was approved by Regional Ethics Committee. Before undergoing to the OIAT, written consent was obtained from each patient.

The OIAT was performed in the morning in patients fasting from the evening before. 60 milligrams of elemental iron belonging to FERALGINE™ was acutely administrated in each patient [23]. Serum iron was evaluated at baseline (T0) and after 2 h (T1) from the oral iron ingestion. Additionally, they were asked not to eat or drink before the second venous blood sample had been drawn. Blood samples were drawn between 8 and 11 a.m.

Statistical analyses were conducted using SPSS 21.0 software. We analysed the mean oral iron absorption in patients with CD and in those without CD using a t-test. Results were considered statistical significant for a *p*-value < 0.05.

## 3. Results

Clinical and laboratory findings of the patients are shown on Table 1.

**Figure 1:**
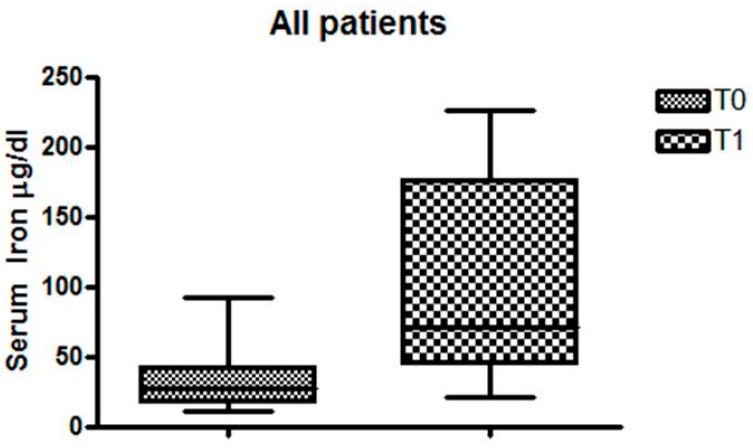
Serum iron levels in all patients.

The mean value of Hb in Group A and Group B was, respectively, 11.07 g/dL and 10.8 g/dL (*p* > 0.05). The OIAT was well tolerated in all patients. There was a clear improvement in iron serum in all patients (T0 = 31.30 µg/dL vs. T1 = 105.3µg/dL, *p* < 0.0001), as can be seen in Figure 1.

**Figure 2:**
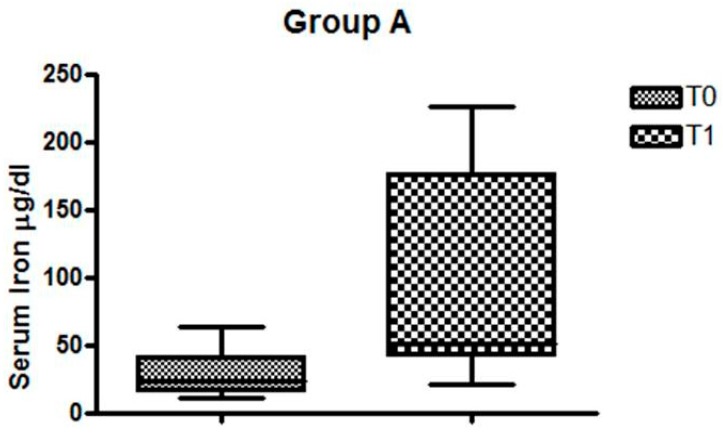
Changes in serum iron levels in celiac disease (CD) patients with iron deficiency anemia (IDA).

In Group A, serum iron before and after Feralgine^TM^ was, respectively, T0 = 28.21 µg/dL and T1 = 94.14 µg/dL (*p* = 0.004), as can be seen in Figure 2.

**Figure 3:**
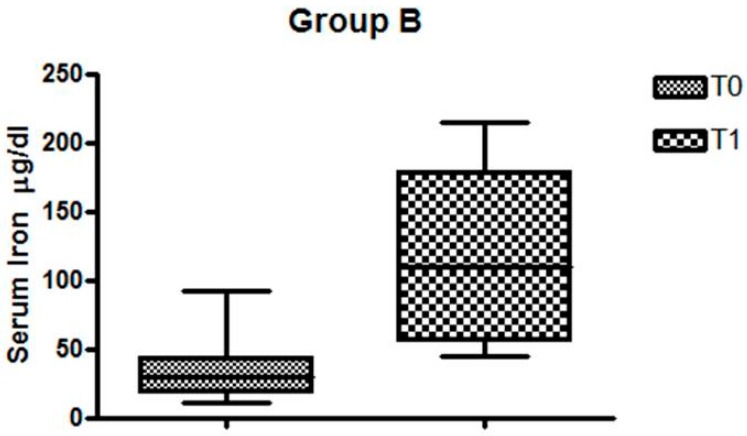
Changes in serum iron levels in IDA patients without CD.

In Group B, the serum iron before and after Feralgine^TM^ was, respectively, T0 = 34.91µg/dL and T1 = 118.83 µg/dL (*p* = 0.0003), as can be seen in Figure 3.

The relationship between the severity of iron deficiency anemia and the absorption of iron showed that patients with severe anemia (Hb< 10 g/dL) have an higher increase in serum iron after the OIAT (about nine times) than mild/moderate forms of anemia (TO: 12 µg/dL and 35.76 µg/dL vs. T1 109.2 µg/dL and 104.66 µg/dL in severe anemia and mild/moderate anemia, respectively).

## 4. Discussion

This was the first study that showed an evident increase of serum iron after the administration of a new kind of FBC in patient with CD. Previous studies suggested that FBC is equally or better absorbed than ferrous sulfate in patients with IDA, with a lower dosage [14,15,16]. In particular, our results confirmed the good absorption of Feralgine^TM^ in IDA previously reported by Rondinelli et al. [24]. These authors studied the OIAT with Feralgine^TM^ in 14 adults with IDA, showing an increase of mean serum iron from 11.21 ± 10.66 to 111.00 ± 51.56 µg/dL. Our results, without a control group, were equally or better than those expected with ferrous sulphate according to the literature data.

The OIAT is a very useful and clear test to evaluate the intestinal absorption of iron through a measurable plasma iron increase in the hours following a single dose of an oral iron preparation [21]. Forgotten for many years, it has recently been proposed again. As is typical for IDA patients, the OIAT is commonly performed by administrating 200 mg of ferrous sulphate, equivalent to 65 mg of elemental iron [23]. Andersen et al. demonstrated that the OIAT performed with ferrous or ferric iron drops showed very different results, with a clear increase of plasma iron concentration after the ingestion of ferrous sulfate iron [25].

In literature, there have been some reports concerning the OIAT in health subjects or in patients with IDA. To the best of our knowledge, the OIAT has never been studied for detecting ferrous sulfate absorption in celiac patients with IDA [19,26]. Our findings show new evidence for the clear efficacy of this new kind of iron in CD patients. Treatment with oral iron supplements in CD is usually ineffective, so CD is frequently diagnosed in patients suffering from IDA that is resistant to oral iron treatment. Only Islam MS et al., performing the OIAT with ferrous sulfate in 238 women with IDA, identified six patients with CD that clearly showed an impairment of iron absorption [27]. Recently, we performed the OIAT with FBC in children with newly diagnosed CD with the characteristic intestinal lesions; we clearly demonstrated that this formulation may be well-absorbed without side effects [28].

The present study provides evidence that Feralgine^TM^ are not only well absorbed in non-celiac IDA patients but are equally absorbed in celiac patients with an overt disease.

The mechanism underlying the good absorption of Feralgine^TM^, as well as FBC in CD with overt mucosa lesions, remains unclear. Many studies have shown that aminoacid-chelated iron is better absorbed than inorganic iron. In particular, several studies agree with the fact that glycine-chelated iron is better absorbed and faster utilized than ferrous sulphate [15]. This might indicate that the iron absorption mechanism is different. At the intestinal mucosa level, non-heme iron absorption is mediated by the divalent metal ion transporter 1 (DMT1) [29,30]. Mucosa lesions in CD induce a reduction of DMT1, which may explain why iron is less absorbed [31]. Recent studies in pigs showed that FBC increases transcript expression of DMT1, the PepT1, and the heme-iron transporter. As such, it may be suggested that FBC may be absorbed as heme-iron via the PepT1. Furthermore, as there is some evidence that ferrous bisglycinate is intact when taken up by the intestinal cells, it may be suggested that the iron complex might be absorbed irrespective of the presence of DMT1. However, data regarding the absorption process and studies about the mechanism of its high bioavailability are limited. The real reason for the good absorption of FBC and Feralgine^TM^ in celiac patients warrants further studies.

## 5. Conclusions

Our results showed that Feralgine^TM^ is well tolerated and well absorbed, not only in anemic non-celiac patients but also in patients with overt CD at the time of diagnosis. As it is widely assumed that side effects limit compliance to iron treatment, this new iron formulation seems very promising for the treatment of iron deficiency.

## Figures and Tables

**Table 1:** Demographic and laboratory baseline comparison data in all patients.

	MALE	FEMALE	HAEMOGLOBIN ± SD (g/dL)	FERRITIN ± SD (ng/dL)	SERUM IRON ± SD (µg/dL)
CELIAC-IDA	2	12	11.07 ± 1.04	10.44 ± 15.9	28.21 ± 14.9
NON CELIAC-IDA	0	12	10.80 ± 0.9	12.30 ± 13.8	34.91 ± 23.2
